# Scientific Activity Is a Better Predictor of Nobel Award Chances than Dietary Habits and Economic Factors

**DOI:** 10.1371/journal.pone.0092612

**Published:** 2014-03-27

**Authors:** Hideyuki Doi, Alexandre Heeren, Pierre Maurage

**Affiliations:** 1 Institute for Sustainable Sciences and Development, Hiroshima University, Higashi-Hiroshima, Japan; 2 Laboratory for Experimental Psychopathology, Psychological Sciences Research Institute, Université Catholique de Louvain, Louvain-la-Neuve, Belgium; Max Planck Society, Germany

## Abstract

Several recent studies have described a strong correlation between nutritional or economic data and the number of Nobel awards obtained across a large range of countries. This sheds new light on the intriguing question of the key predictors of Nobel awards chances. However, all these studies have been focused on a single predictor and were only based on simple correlation and/or linear model analysis. The main aim of the present study was thus to clarify this debate by simultaneously exploring the influence of food consumption (cacao, milk, and wine), economic variables (gross domestic product) and scientific activity (number of publications and research expenditure) on Nobel awards. An innovative statistical analysis, hierarchical partitioning, has been used because it enables us to reduce collinearity problems by determining and comparing the independent contribution of each factor. Our results clearly indicate that a country's number of Nobel awards can be mainly predicted by its scientific achievements such as number of publications and research expenditure. Conversely, dietary habits and the global economy variable are only minor predictors; this finding contradicts the conclusions of previous studies. Dedicating a large proportion of the GDP to research and to the publication of a high number of scientific papers would thus create fertile ground for obtaining Nobel awards.

## Introduction

Nobel Prizes are among the most famous and most prominent scientific awards worldwide. In view of the international prestige related to these prizes, determining the key variables that influence the number of Nobel Prizes obtained is a key question in the orientation of countries' scientific policies. In 2012, a paper published in the *New England Journal of Medicine* [1] reported a strong correlation between chocolate consumption and the number of Nobel Prizes obtained in 23 countries. The author explained that this surprising result was due to the beneficial effect of cocoa-flavanols on cognition; this led to the proposal that increasing chocolate consumption at the population level might increase a country's chances of obtaining Nobel Prizes. We have already demonstrated [2] that flavanol concentration does not fully explain this result, as no correlation was found between the number of Nobel laureates and other flavanol-rich nutriments (e.g., tea and wine). Moreover, we underscored that, as correlation never implies causation, the simple link reported in the initial paper cannot be interpreted as implying a causal relation between chocolate consumption and Nobel awards, and that hidden factors might moderate this link.

Despite warnings on the danger of over-interpreting correlations, this initial observation was widely broadcasted by popular media, was taken as fact by several scientific publications [3], [4], and was accepted and extended in a letter published in *Nature* [5]. Moreover, to capitalize on this result, several recent papers have tried to identify other dietary habits that might predict the number of Nobel laureates. For example, significant positive correlations have been reported between Nobel laureates and milk consumption [6], [7]. It has also been postulated that global economic factors might moderate the links between nutritional factors and Nobel awards. In particular, the economic strength of a country, as estimated by the gross domestic product (GDP), is significantly correlated with both chocolate consumption and Nobel awards, and might thus explain the links observed [2], [8]. Nevertheless, as these results were based on a simple correlational approach, they all have the same limitations than the initial report, and they were thus unable to clearly identify the predictors of Nobel awards.

Obtaining Nobel awards clearly constitutes a crucial challenge for nations worldwide, as they are a significant determinant of a country's prestige and a reliable index of the efficiency of scientific policy. The question of the main variables that contribute to the obtaining of Nobel awards is therefore crucial, but the methodology used in the studies mentioned above is highly questionable and does not allow the precise determination of the respective weight of each factor. Centrally, these earlier studies featured two main shortcomings that caution against drawing any strong conclusions from the results obtained.

First, they each focused on one specific explanatory factor and ignored the central variables that might predict the number of Nobel awards obtained. In particular, beyond dietary habits and GDP, an obvious factor that might affect Nobel awards obtained is the country's level of scientific funding and activity. Indeed, a lay hypothesis would be that the more a country invests in scientific research, the more its researchers will publish decisive results, which, in turn, would increase its scientific renown and its Nobel award chances. The countries with the highest research activity (such as higher research expenditures and larger numbers of publications) should thus be those with more potential Nobel awardees. In this paper, we hypothesize that the level of research activity in a country is important in predicting the number of Nobel laureates, especially for scientific fields.

Second, previous studies did not consider the collinearity problem among the explanatory factors in the statistical approaches they used. Indeed, these studies only used correlation and multiple regression analyses, which can be seriously affected by multicollinearity between the explanatory variables [9]–[10], as notably illustrated by the strong correlations between GDP and dietary consumption. Nonetheless, collinearity problems can be effectively alleviated using an analytical method called hierarchical partitioning [11]–[13]. Hierarchical partitioning reduces collinearity problems by determining the independent contribution of each explanatory variable to the response variable and separates it from the joint contribution that results from correlations with other variables [11]. This allows the contributions of the covariates in explaining the predicted variables to be ranked independently of the others' covariates. Therefore, we use hierarchical partitioning to control for the collinearity problem and to estimate the effects of explanatory factors on the number of Nobel laureates in each country.

The main aim of this study is thus to use an innovative statistical technique, hierarchical partitioning, to clarify the debate concerning the crucial predictors of a country's number of Nobel awards. While earlier studies were based on simple correlations and focused on a specific predictor, the central strength of the present study is to simultaneously explore and compare the influences of a large range of explanatory factors, including some mentioned earlier (food consumption and GDP) and others that are totally unexplored (publication level and research expenditure).

## Materials and Methods

### Collecting Nobel Prize Data

All Nobel Prize winners between 1901 and 2013 in all disciplines (N = 851) were included. Data were obtained from Wikipedia (http://en.wikipedia.org/wiki/List_of_countries_by_Nobel_laureates_per_capita, Accessed 14 Oct. 2013). Prizes are allocated to the country/countries stated on the winner's biography on the website of the Nobel Prize committee (www.nobelprize.org). When the website mentions multiple countries in relation to a prize winner (country of birth; country of citizenship; country of residence at the time of award) each of those countries is credited as having won the prize. Where a Nobel Prize has multiple winners, the country (or countries) of each winner are credited. Prizes which were declined by the winner are included. Prizes received by international organizations are not allocated to countries (http://en.wikipedia.org/wiki/List_of_countries_by_Nobel_laureates_per_capita). The only Nobel Prize awarded to a country-based organization has been given to the Grameen Bank (Bangladesh), but as this prize was also awarded to its funder (Muhammad Yunus), it has already been counted as a prize awarded to Bangladesh. All the other Nobel Prizes awarded to organizations have been attributed to international ones like EU, IPCC, and have thus not been included in our statistical analyses.

In total, 71 countries had at least one laureate. In order to control for each country's population and in line with earlier studies [1], the total population of each country was obtained from “Total Population – Both Sexes” in the 2012 revision of *World Population Prospects* (http://esa.un.org/), which is provided by the United Nations Department of Economic and Social Affairs, Population Division, Population Estimates and Projections Section. The number of Nobel laureates per 10 million people was computed for each country (N = 220). Natural science Nobel Prizes and other Nobel Prizes might be predicted differently; therefore, two separate analyses were performed: one focusing on Nobel laureates in natural sciences (i.e., physics, chemistry, physiology, and medicine) and one for all disciplines (i.e., natural sciences plus literature, economics, and peace).

### Collecting the Explanatory Factor Data

Explanatory data predicting the number of Nobel laureates in each country were collected. First, the indicators of economic and scientific activity were collected from the World Bank Database's website (http://data.worldbank.org, Accessed 13 Sep. 2013), and the following data were collected. (1) GDP per capita. GDP is the sum of the gross value added by all resident producers in the economy plus any product taxes and minus any subsidies not included in the value of the products. This has been calculated without making deductions for the depreciation of fabricated assets or for the depletion and degradation of natural resources. GDP per capita has been calculated as GDP divided by the midyear population in each country. (2) The percentage of GDP dedicated to research and development expenditure (hereafter, “research expenditure”). Research and development covers basic research, applied research, and experimental development. Expenditures for research and development include current and capital expenditures (both public and private) on creative work undertaken systematically to increase knowledge, including the knowledge of humanity, culture, and society, and the use of this knowledge for new applications. (3) The number of research articles in peer-reviewed journals (hereafter, “number of publications”). This includes journal articles published in the following fields: physics, biology, chemistry, mathematics, clinical medicine, biomedical research, engineering and technology, and earth and space science. The data for GDP, research expenditure, and publications were available from 1961 to 2012, from 1996 to 2010, and from 1986 to 2009, respectively.

Next, in order to explore the predictive value of nutritional habits in line with the factors explored in earlier studies, the consumption of cacao beans, milk (including other milk products such as butter), and wine per capita per year were collected for each country from the Food and Agriculture Organization of the United Nations' website (FAO, http://www.fao.org, Accessed 13 Sep. 2013). These data were available from 1961 to 2009. Consumption was recorded in kilograms per capita per year, and the mean values from 1961 to 2009 were computed for the explanatory factors in the statistical analysis.

Importantly, while previous studies only used economic and food consumption data for a particular year (most frequently for very recent years), the present study was based on the computation of the mean values for each explanatory factor during the data collection period. This method allows us to reduce the discrepancy between the time periods considered for Nobel Prizes (1901–2013) and those for explanatory factors, as the time period for most of the factor data, which begins in the 1960s, includes less than half of the Nobel Prizes awarded. In total, 220 countries and regions have been used for the following analyses, including the countries and regions that did not receive any Nobel Prize (http://www.fao.org).

### Statistical Analysis

All statistical analyses were performed using R version 2.15.2 [14]. Hierarchical partitioning [11] was performed to estimate the independent explanatory capacity of each factor on the number of all Nobel laureates and natural science laureates per capita. The process of hierarchical partitioning involves the computation of the increase in the goodness of fit (here, the R^2^ value) of all models with a particular variable compared with the equivalent model without that variable, and averaging the improvement in the fit across all possible models with that predictor. [11]. Thus, hierarchical partitioning estimates the independent and joint contributions of each explanatory factor with all other factors separately. We used the independent contribution (R^2^) of each explanatory factor for the results. For hierarchical partitioning, the “hier.part” function in the “hier.part” package was used [15]. Gaussian distribution was used as the family distribution for hierarchical partitioning. To evaluate whether each factor accounted for a greater unique variation than expected by chance, a randomization test (1,000 randomizations) was conducted using the “rand.hp” function in the “hier.part” package. The significance of the randomization test was based on an upper confidence limit of 0.95 (Z≥1.65). This approach has been successfully used to deal with multicollinearity among explanatory variables, especially in ecological data [11]–[13]. Crucially, this method appears particularly appropriate for the present study, as it has been recently demonstrated that the hierarchical partitioning approach is particularly well-suited when less than nine explanatory factors are used to determine the ranking of covariate importance [13].

Before performing the analysis, the normality of each factor was checked using the Shapiro-Wilk normality test, which revealed that all factors deviated significantly from the normal distribution (p<0.001 for all factors). To take this into account, the values of the factors were transformed using the log10 (x+1) equation to perform hierarchical partitioning. Pearson's correlation coefficient was also used to check the collinearity.

## Results

**Figure 1:**
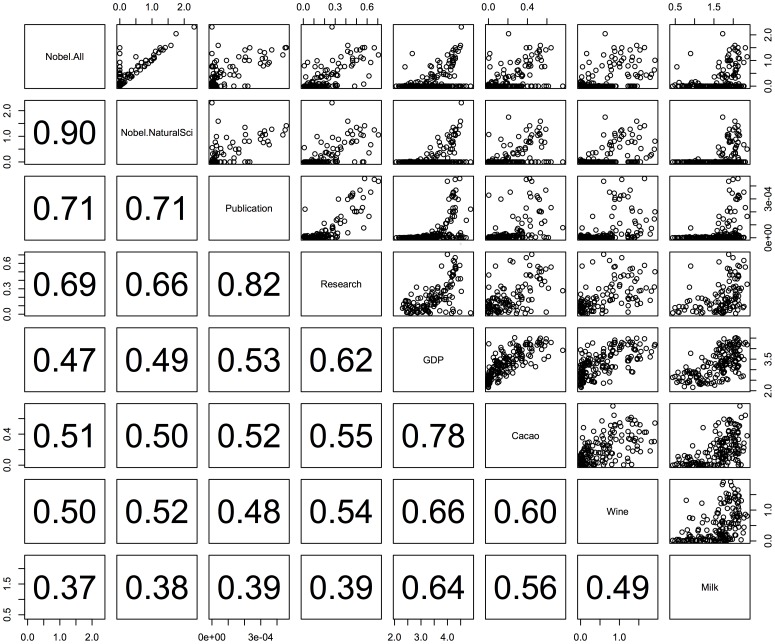
Relations between the numbers of Nobel laureates per 10 million people and the explanatory factors. The relations are given for all Nobel categories (“Nobel.All”) and for natural sciences' Nobel laureates (Nobel.NatSci). The factors are Research Expenditure (% of GDP), Publication (number of scientific articles), GDP, Cacao (cacao bean consumption per capita), Wine (wine consumption per capita), and Milk (milk consumption per capita). The numbers in the lower boxes indicate the Pearson's correlation coefficients; all the coefficients are significant (p<0.001).

The results of the correlation analysis among the factors are represented in Figure 1. Because of the large sample size of the data, all the Pearson's correlation coefficients were significant (p<0.001). The numbers of all Nobel laureates and natural science laureates were strongly correlated (r = 0.90), indicating that the number of all Nobel laureates was mainly derived from the Nobel laureates for natural science.

**Figure 2:**
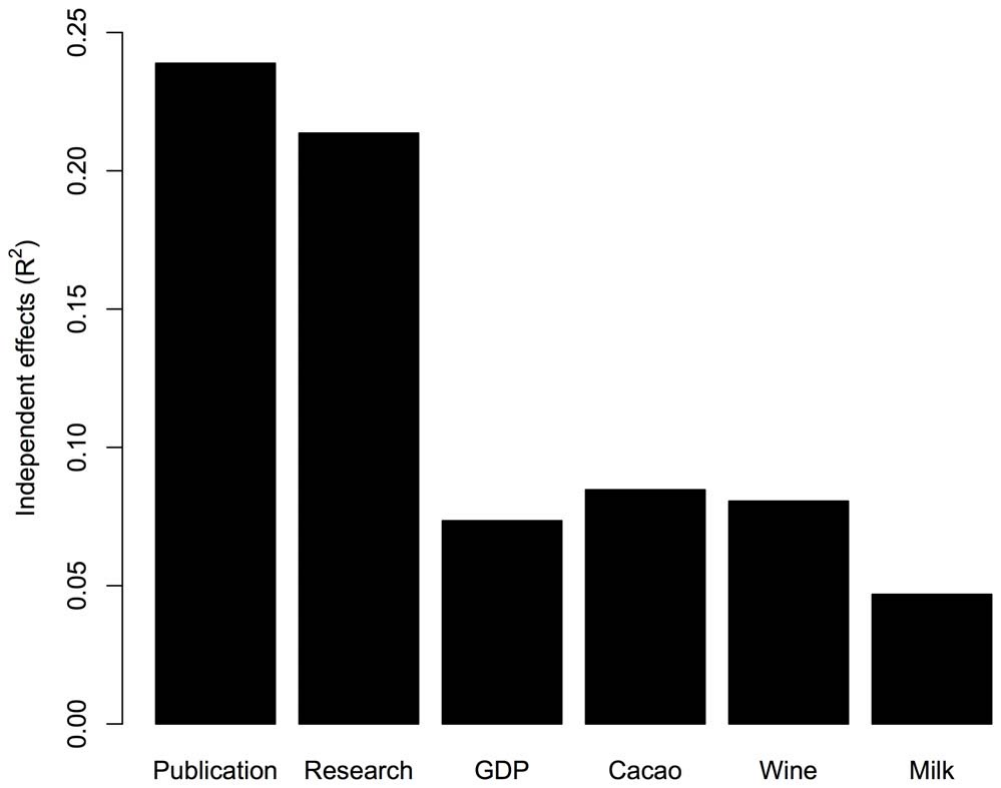
The independent effect (R^2^) of each factor on the number of Nobel laureates for all Nobel categories. The independent effects are analyzed by hierarchical partitioning.

**Figure 3:**
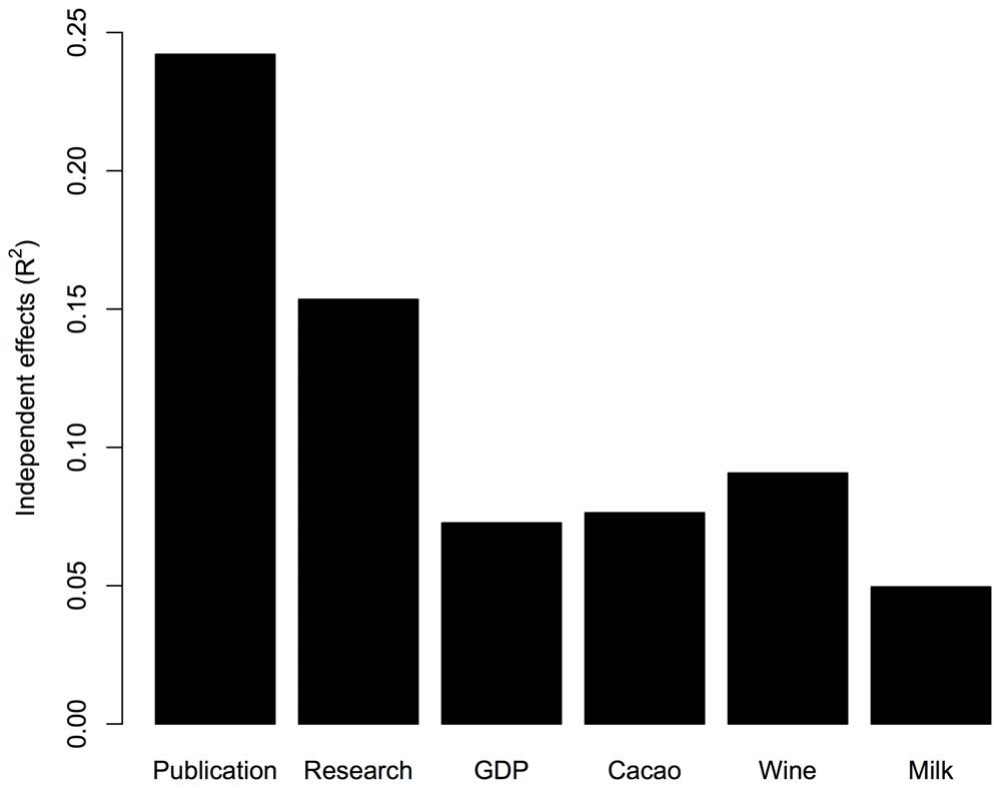
The independent effect (R^2^) of each factor on the number of Nobel laureates for natural sciences. The independent effects are analyzed by hierarchical partitioning.

Hierarchical partitioning for the number of all Nobel laureates is depicted in Figure 2, which shows that Publication Number had the highest independent contribution among the factors, followed by Research Expenditure (% of GDP), while other factors (GDP, Cacao, Milk, and Wine) had lower contributions (R^2^<0.1). The specific analysis for natural sciences laureates (Figure 3) produced similar results, as the hierarchical partitioning showed that Publication Number had the highest independent contribution, followed by Research Expenditure, while other factors had lower contributions (R^2^<0.1).

The randomization test (n = 1,000) for hierarchical partitioning indicated that five factors predicted the number of all Nobel laureates at a higher degree than expected by chance, namely Publication (Z-score = 16.09), Research (8.77), GDP (5.29), Cacao, (3.53), Wine (3.35), and Milk, which was not a significant predictor (1.55). Similarly, these five factors significantly predicted the number of Nobel laureates for natural science (Z-scores: Publication, 14.41; Research, 4.98; GDP, 4.30; Cacao, 3.15; Wine, 3.67); again, Milk (1.63) was not a significant predictor.

## Discussion

Nobel awards are a central indicator of a country's scientific achievement, which includes the number of publications and research expenditure. Therefore, the exploration of the key factors that might predict the number of Nobel laureates obtained is a very important question. This debate has recently been reopened by the publication of several papers [1], [2], [6]–[8] that have postulated the influence of dietary habits (the consumption of chocolate, milk, and wine) or global economic variables (GDP) on the obtaining of Nobel Prizes. While the great merit of these studies was to offer new insight into this challenging issue, their methodological and logical shortcomings hampered the clear identification of the main factors that predict the number of Nobel awards obtained and their respective influences. The main contribution of the present study was, therefore, to use a validated statistical analysis, hierarchical partitioning, to overcome these limitations by simultaneously exploring a large range of variables that potentially affect Nobel awards. The identification of the independent influence of each predictor led to two crucial results.

First, our results clearly showed that the number of publications and research expenditure, which had been totally ignored in earlier studies, are key factors in predicting Nobel award chances, as they make the highest independent contributions in predicting a country's number of Nobel laureates for natural science as well as for Nobel laureates irrespective of discipline. The number of publications and research expenditure are reliable indicators of a country's research activity level, and our hypothesis that these factors would effectively predict the number of Nobel laureates across countries is thus confirmed. While this result appears quite logical, as it is not surprising that dedicating a larger part of the GDP to research funding and publishing many scientific articles would constitute a more fertile ground for scientific achievement and, consequently, for obtaining Nobel awards, this link had not been investigated earlier. Previous studies based on simple correlations suggested that global economic factors might play a central role in predicting the number of Nobel awards obtained [2], [8], but the present results clarify this link by showing that it is the proportion of the GDP dedicated to research rather than the richness of the country (that is, the GDP itself) that is the central determinant of Nobel award chances.

Second, hierarchical partitioning applied to the data of 220 countries clearly showed that nutritional habits, which were the central focus of earlier studies [1], [6], [7], do not constitute a key predictor of Nobel awards. Using a limited number of countries, these previous studies explored some correlations between chocolate, milk, and wine consumption and Nobel awards, concluding that food might play a crucial role in obtaining Nobel awards. More specifically, recent studies published in renowned journals [1], [5] proposed that increasing chocolate consumption might be an efficient way to improve cognition at the individual level and the chances of Nobel awards at the country level. Despite the amazing extent to which this proposal was broadcasted in popular and scientific media, our results obviously refute this hypothesis by showing that nutritional factors have a very modest influence on predicting the number of Nobel laureates.

It should be mentioned that this study, in line with previous ones in the field, used a country-level dataset to predict the number of Nobel laureates; however, the personal dietary habits, publication levels, and scientific funding of the actual Nobel laureates remain unknown. Further study might thus complement the present one by obtaining the personal-level data of Nobel laureates to directly explore the influence of these predictors. Our analysis used the mean values of the explanatory factors, such as GDP, but during 1901–2013, the economy, dietary habits and scientific activity have been drastically modified. In this study, we used the total number of Nobel laureates in the countries as an index of scientific success, but future studies should use time-series analysis for scientific success to explore the effects of long-term changes on these factors.

The Nobel Prize data which have been used in this study raise some concerns that should be underlined. First, Nobel Prizes are not the only index of scientific achievement and their attribution is influenced by non-scientific factors (e.g., political or diplomatic reasons). Second, the researchers who did not obtain the Nobel Prize for their country of birth cannot be considered in the analysis. In order to take this into account, when the Nobel Prize winner had moved during his/her scientific career, a Nobel Prize for each country concerned (e.g. country of birth; country of citizenship; country of residence at time of award) should be included in the analyses. Moreover, the scientific reputation of a country is also related to its ability to catch researchers from other countries, and we thus believe that the Nobel Prizes received by countries via foreign or immigrant researchers is also related to this country's scientific policies. Further study needs to explore the influence of these variables on the scientific achievements of the countries.

In conclusion, our hope is that the present results, which precisely identify the key predictors of Nobel award chances at the country level, will clarify the debate concerning the roles played by nutritional, economic, and scientific factors on the awarding of Nobel Prizes. In particular, the very modest influence played by dietary habits in our analysis should encourage future studies to avoid drawing strong conclusions and giving nutritional advice based on simple correlations observed between nutriment consumption and cognitive ability.
